# Improved Stabilization Method for Lurie Networked Control Systems

**DOI:** 10.1155/2014/789398

**Published:** 2014-04-17

**Authors:** Hong-Bing Zeng, Lei Ding, Shen-Ping Xiao, Fei Yu

**Affiliations:** ^1^School of Electrical and Information Engineering, Hunan University of Technology, Zhuzhou 412007, China; ^2^School of Information Science and Engineering, Jishou University, Jishou 416000, China; ^3^Jiangsu Provincial Key Laboratory for Computer Information Processing Technology, Soochow University, Soochow 215006, China

## Abstract

The problem of stabilization of Lurie networked control systems (NCSs) is investigated in this paper. The network-induced delays in NCSs are assumed to be time-varying and bounded. By utilizing a reciprocally convex technique to consider the relationship between the network-induced delay and its varying interval, a new absolute stability condition is derived in terms of linear matrix inequalities (LMIs). Based on the obtained condition, an improved cone complementary linearisation (CCL) iteration algorithm is presented to design a state feedback controller. The effectiveness of the proposed method is verified by a numerical example.

## 1. Introduction

The so-called networked control system (NCS) is a spatially distributed system, wherein the control loop is closed through a real-time network. Compared with the traditional point-to-point feedback control system, NCS provides many advantages, such as low cost, flexibility of operation, simple diagnosis and maintenance, and high reliability. Thus, NCSs have a wide application in practical systems during the last decades. In an NCS, network-induced delays including those produced in the signal transmission from controller to actuator and sensor to controller cannot be avoided while exchanging data among devices connected to the shared network medium, which inevitably lead to the degradation of the system performance and even destabilize the system [1–10]. Therefore, it is very important to design a valid controller to assure the stability of NCS within a maximum allowable delay bound (MADB) [11].

In [8], some stability conditions under an assumption that the networked-induced delay is less than the sampling period were derived based on sample-date method. A new model that the networked-induced delay is larger than the sampling period was presented in [12], where some new conditions are derived to stabilize an NCS. The similar idea is also employed to investigate the *H*_*∞*_ control problem in [13]. In addition, by considering the relationship between the network-induced delay and its varying interval, some improved results for networked control system are proposed in [14]. Nevertheless, all the above literatures are focused on the linear NCSs, while the nonlinear phenomenon is essential and universal in practical system. Therefore, it is more significant to investigate nonlinear NCSs. Recently, a special nonlinear NCS, Lurie NCS, has been investigated in [15]. By retaining a useful term ignored in the derivative of Lyapunov-Krasovskii in [15] and employing free-weighting matrix method, some improved conditions are proposed in [16]. However, redundant free-weighting matrices are introduced in [16], which inevitably increase the amount of computation.

To get a networked state feedback controller gain, a parameter-tuning method was presented in [12], in which some scalars are introduced. Nevertheless, it is very difficult to find the optimum parameters. In [15], an inequality approach was proposed, which is simple and convenient since there is no need to adjust any parameters, but the obtained result is considerably conservative. Based on the cone complementary linearisation (CCL) algorithm [17], Moon et al. proposed an LMI-based iterative algorithm to design a delay-dependent state feedback stabilization controller, while the stopping conditions for iteration are very strict [18]. With a new stopping condition, an improved (CCL) algorithm is proposed in [14]. However, a great amount of computation is needed in the iteration process in [14, 16]. This motivates the present study.

In this paper, a new absolute stability criterion for Lurie NCSs is firstly established by employing a reciprocally convex technique to consider the relationship between network-induced delay and its upper bound. Then, with a new iteration condition, a state feedback controller design method is presented based on the CCL algorithm, which needs less number of iterations in computing the MADB and the corresponding controller. The effectiveness of the proposed method is verified by a numerical example.

*Notation*. Throughout this paper, *P* > 0 means that the matrix *P* is positive definite; *I* represents an appropriately dimensioned identity matrix, the superscripts “−1” and “*T*” stand for the inverse and transpose of a matrix, respectively, *R* ^*n*^ denotes the *n*-dimensional Euclidean space, *R* ^*n*×*m*^ is the set of all *n* × *m* real matrices, diag⁡{⋯} denotes a block-diagonal matrix, and the symmetric terms in a symmetric matrix are denoted by ∗; for example, $\left[\begin{array}{cc} X & Y\\ {}* & Z \end{array}\right]=\left[\begin{array}{cc} X & Y\\ Y^{T} & Z \end{array}\right]$.

## 2. Problem Statement

Consider the following Lurie control system:

(1) $$\begin{array}{c} \dot{x}\left(t\right)=Ax\left(t\right)+Bu\left(t\right)+Dw\left(t\right),\\ z\left(t\right)=Cx\left(t\right),\\ w\left(t\right)=-\varphi \left(t,z\left(t\right)\right), \end{array}$$

where *x*(*t*) ∈ *R* ^*n*^ is the state vector and *u*(*t*) ∈ *R* ^*m*^ is the controlled input vector. *z*(*t*) ∈ *R* ^*p*^ is the measured output. *A*, *B*, *C*, and *D* are constant matrices with appropriate dimensions. *φ*(*t*, *z*(*t*)) is a memoryless, nonlinear function that is piecewise continuous in *t*, is globally Lipschitz in *z*(*t*), *φ*(*t*, 0) = 0, and satisfies the following condition for all *t* ≥ 0 and for all *z*(*t*) ∈ *R* ^*p*^:

(2) $$\begin{array}{c} \varphi^{T}\left(t,z\left(t\right)\right)\left[\varphi \left(t,z\left(t\right)\right)-\Theta z\left(t\right)\right]\leq 0, \end{array}$$

where Θ is a real diagonal matrix. The set of all functions satisfying the above sector condition is denoted by *φ*(·) ∈ *F*[0, Θ].

For the convenience of investigation, the following assumption is introduced [13–15].

**Assumption 1**

(i) The considered NCS consists of a time-driven sensor, event-driven controller, and event-driven actuator, which are all connected through a control network. The calculated time-delay is viewed as a part of the network-induced delay yielded from controller to actuator.

(ii) The controller always uses the most recent data and discards the old data. When old data arrive at the controller, it is treated as packet loss.

(iii) The real input realized through a zeroth order holding in (1) is a piecewise constant function.

Under Assumption 1, following the similar technique employed in [13–15], the closed-loop system with memoryless state feedback controller can be represented as

(3) $$\begin{array}{c} \dot{x}\left(t\right)=Ax\left(t\right)+BKx\left(t-\tau_{k}\left(t\right)\right)+Dw\left(t\right),\\ x\left(t\right)=\phi \left(t\right),\;t\in \left(t_{0}-\eta ,t_{0}\right), \end{array}$$

where 0 ≤ *τ*_*k*_(*t*) ≤ *η* and *ϕ*(*t*) is the initial condition function, which is a continuous and differentiable vector-valued one.

In the sequel, we introduce two lemmas, which are indispensable in deriving our main results.

**Lemma 2 (see [19])**

Let *M* = *M* ^*T*^ > 0 be a constant real *n* × *n* matrix, and suppose $\dot{x}:[-h,0]\mapsto R^{n}$ with *h* > 0 such that the subsequent integration is well defined. Then, one has

(4) $$\begin{array}{c} -h\overset{t}{\underset{t-h}{\int }}{\dot{x}}^{T}\left(s\right)M\dot{x}\left(s\right)ds\leq \zeta^{T}\left(t\right)\left[\begin{array}{cc} -M & M\\ {}* & -M \end{array}\right]\zeta \left(t\right), \end{array}$$

where *ζ*(*t*) = col {*x*(*t*), *x*(*t* − *h*)}.

**Lemma 3 (see [20])**

Let *H*_1_, *H*_2_,…, *H*_*N*_ : *R* ^*n*^ ↦ *R* be given finite functions, and they have positive values for arbitrary value of independent variable in an open subset *M* of *R* ^*n*^. The reciprocally convex combination of *H*_*i*_  (*i* = 1,2,…, *N*) in *M* satisfies

(5) min⁡    ∑i=1N1λiHi(t)=∑i=1NHi(t)+max⁡∑i=1N ∑j=1, j≠iNGi,j(t)
subject  to
 {λi>0,∑i=1Nλi=1,Gi,j(t):Rn↦R,        Gj,i(t)=Gi,j(t),[Hi(t)Gi,j(t)Gi,j(t)Hj(t)]≥0}.

## 3. Main Results

In this section, we first discuss the case of Lurie NCS (3) with a given gain *K*. By using Lyapunov-Krasovskii method, the following stability criterion is obtained.

**Theorem 4**

Given a scalar *η* > 0 and a controller gain matrix *K*, the closed-loop system (3) with nonlinear function *φ*(·) ∈ *F*[0, Θ] is absolutely stable if there exist matrices *P* = *P* ^*T*^ > 0, *Q* = *Q* ^*T*^ > 0, and *Z* = *Z* ^*T*^ > 0 and an appropriately dimensional matrix *G*, such that the following matrix inequalities hold:

(6) $$\begin{array}{c} {\overset{-}{\Phi }}_{1}=\left[\begin{array}{ccccc} \Phi_{11} & \Phi_{12} & G & PD-C^{T}\Theta^{T} & \eta A^{T}Z\\ {}* & \Phi_{22} & Z-G & 0 & \eta K^{T}B^{T}Z\\ {}* & * & -Q-Z & 0 & 0\\ {}* & * & * & -2I & \eta D^{T}Z\\ {}* & * & * & * & -Z \end{array}\right]<0, \end{array}$$

(7) $$\begin{array}{c} {\overset{-}{\Phi }}_{2}=\left[\begin{array}{cc} Z & G\\ {}* & Z \end{array}\right]>0, \end{array}$$

where

(8) Φ11=PA+ATP+Q−ZΦ12=PBK+Z−GΦ22=−2Z+G+GT.

**Proof**

Choose a Lyapunov-Krasovskii functional candidate to be

(9) V(xt)=xT(t)Px(t)+∫t−ηtxT(s)Qx(s)ds+η∫−η0∫t+θtx˙T(s)Zx˙(s)ds dθ,

where *P* = *P* ^*T*^ > 0, *Q* = *Q* ^*T*^ > 0, and *Z* = *Z* ^*T*^ > 0 are to be determined.

Calculating the derivative of *V*(*x*_*t*_) along the solutions of system (3) yields

(10) V˙(xt)=2xT(t)Px˙(t)+xT(t)Qx(t)−xT(t−η)Qx(t−η)+η2x˙T(t)Zx˙(t)−η∫t−ηtx˙T(s)Zx˙(s)ds.

According to Lemmas 2 and 3, it can be deduced that

(11) −η∫t−ηtx˙T(s)Zx˙(s)ds  =−η∫t−τk(t)tx˙T(s)Zx˙(s)ds   −η∫t−ηt−τk(t)x˙T(s)Zx˙(s)ds  ≤−ητk(t)[x(t)x(t−τk(t))]T   ×[Z−Z∗Z][x(t)x(t−τk(t))]   −ηη−τk(t)[x(t−τk(t))x(t−η)]T   ×[Z−Z∗Z][x(t−τk(t))x(t−η)]  ≤−[x(t)−x(t−τk(t))x(t−τk(t))−x(t−η)]T[ZG∗Z]   ×[x(t)−x(t−τk(t))x(t−τk(t))−x(t−η)]  =ξ1T(t)[−ZZ−GG∗−2Z+G+GTZ−G∗∗−Z]ξ1(t)

for $\left[\begin{array}{cc} Z & G\\ {}* & Z \end{array}\right]>0$, where *ξ*_1_^*T*^(*t*) = [*x*(*t*)^*T*^ *x*(*t* − *τ*_*k*_(*t*))^*T*^ *x*(*t* − *η*)^*T*^].

From (2), we have

(12) $$\begin{array}{c} 0\leq -2w^{T}\left(t\right)w\left(t\right)-2w^{T}\left(t\right)\Theta Cx\left(t\right). \end{array}$$

Adding the right sides of (12) into (10) and applying (11) yield

(13) V˙(xt)≤2xT(t)Px˙(t)+xT(t)Qx(t)−xT(t−η)Qx(t−η)+η2x˙T(t)Zx˙(t)−η∫t−ηtx˙T(s)Zx˙(s)ds−2wT(t)w(t)−2wT(t)ΘCx(t)≤ξ2T(t)[Φ1+η2Φ2TZΦ2]ξ2(t),

where

(14) Φ1=[Φ11Φ12GPD−CTΘT∗Φ22Z−G0∗∗−Q−Z0∗∗∗−2I]Φ2=[ABK0D]ξ2(t)=[xT(t)xT(t−τk(t))xT(t−η)wT(t)]T.

Thus, if Φ_1_ + *η* ^2^Φ_2_^*T*^ *Z*Φ_2_ < 0, which is equivalent to (6) by virtue of Schur complements [21], $\dot{V}(x_{t})<-\varepsilon {||x(t)||}^{2}$ for a sufficiently small *ε* > 0. Hence, system (3) is absolutely stable. This completes the proof.

**Remark 5**

In the proof in [15], the term $-\int_{t-\eta }^{t}{\dot{x}}^{T}(\alpha )R\dot{x}(\alpha )d\alpha$ is enlarged to $-\int_{t-\tau_{k}(t)}^{t}{\dot{x}}^{T}(\alpha )R\dot{x}(\alpha )d\alpha$. In contrast, in the proof of Theorem 4, $-\eta \int_{t-\eta }^{t}{\dot{x}}^{T}(\alpha )Z\dot{x}(\alpha )d\alpha$ is taken to be $-\eta \int_{t-\tau_{k}(t)}^{t}{\dot{x}}^{T}(s)Z\dot{x}(s)ds-\eta \int_{t-\eta }^{t-\tau_{k}(t)}{\dot{x}}^{T}(s)Z\dot{x}(s)ds$. Also, the relationship between *η*/*τ*_*k*_(*t*) and *η*/(*η* − *τ*_*k*_(*t*)) has been taken into account by utilizing a reciprocally convex technique. In addition, compared with the results obtained in [16], less free-weighting matrices are introduced in Theorem 4, which will reduce the amount of computation.

Next, Theorem 4 is extended to design a stabilization controller *K* for system (3).

**Theorem 6**

Given a scalar *η* > 0, the closed-loop system (3) with nonlinear function *φ*(·) ∈ *F*[0, Θ] is absolutely stable if there exist matrices *L* = *L* ^*T*^ > 0, *W* = *W* ^*T*^ > 0, and *R* = *R* ^*T*^ > 0 and any appropriately dimensional matrices *G* and *V*, such that the following matrix inequalities hold:

(15) $$\begin{array}{c} {\overset{-}{\Xi }}_{1}=\left[\begin{array}{ccccc} \Xi_{11} & \Xi_{12} & S & D-LC^{T}\Theta^{T} & \eta LA^{T}\\ {}* & \Xi_{22} & R-S & 0 & \eta V^{T}B^{T}\\ {}* & * & -W-R & 0 & 0\\ {}* & * & * & -2I & \eta D^{T}\\ {}* & * & * & * & -LR^{-1}L \end{array}\right]<0, \end{array}$$

(16) $$\begin{array}{c} {\overset{-}{\Xi }}_{2}=\left[\begin{array}{cc} R & S\\ {}* & R \end{array}\right]>0, \end{array}$$

where

(17) Ξ11=AL+LAT+W−R,Ξ12=BV+R−S,Ξ22=−2R+S+ST.

Moreover, a stabilizing controller gain is given by *K* = *VL* ^−1^.

**Proof**

Pre- and postmultiply ${\overset{-}{\Phi }}_{1}$ in (6) by diag⁡{*P* ^−1^, *P* ^−1^, *P* ^−1^, *I*, *Z* ^−1^} and pre- and postmultiply ${\overset{-}{\Phi }}_{2}$ in (7) by diag⁡{*P* ^−1^, *P* ^−1^}, respectively, and make the following changes to the variables:

(18) $$\begin{array}{c} L≔P^{-1},\;\;R≔P^{-1}ZP^{-1},\;\;V≔KP^{-1},\\ W:=P^{-1}QP^{-1},\;\;S:=P^{-1}GP^{-1}. \end{array}$$

Matrix inequalities (15) and (16) are derived. This completes the proof.

Note that the condition in Theorem 6 cannot be implemented directly by utilizing numerical software due to the existence of the nonlinear term *LR* ^−1^ *L* in (15). To get a networked state feedback controller gain, the following CCL algorithm is employed to solve this nonconvex problem.

Define a new variable *U* such that *U* ≤ *LR* ^−1^ *L*, and replace the condition (15) with

(19) $$\begin{array}{c} {\overset{-}{\Xi }}_{1}=\left[\begin{array}{ccccc} \Xi_{11} & \Xi_{12} & S & D-LC^{T}\Theta^{T} & \eta LA^{T}\\ {}* & \Xi_{22} & R-S & 0 & \eta V^{T}B^{T}\\ {}* & * & -W-R & 0 & 0\\ {}* & * & * & -2I & \eta D^{T}\\ {}* & * & * & * & -U \end{array}\right]<0, \end{array}$$

(20) $$\begin{array}{c} U\leq LR^{-1}L. \end{array}$$

Inequality (20) is equivalent to *U* ^−1^ − *L* ^−1^ *RL* ^−1^ ≥ 0, which can be expressed as

(21) $$\begin{array}{c} \left[\begin{array}{cc} U^{-1} & L^{-1}\\ L^{-1} & R^{-1} \end{array}\right]\geq 0 \end{array}$$

by virtue of Schur complements. Thus, by introducing new variables *P*, *H*, *Z*, the original condition (15) is represented as (19) and

(22) $$\begin{array}{c} \left[\begin{array}{cc} H & P\\ P & Z \end{array}\right]\geq 0,\;\;H=U^{-1},\;\;P=L^{-1},\;\;Z=R^{-1}. \end{array}$$

Then, this nonconvex problem is converted to the following LMI-based nonlinear minimization problem:

 Minimize tr{*LP* + *UH* + *RZ*}
 subject to (16), (19) and
  (23) $$\begin{array}{c} \left[\begin{array}{cc} H & P\\ P & Z \end{array}\right]\geq 0,\;\;\left[\begin{array}{cc} L & I\\ I & P \end{array}\right]\geq 0,\;\;\left[\begin{array}{cc} U & I\\ I & H \end{array}\right]\geq 0,\\ \left[\begin{array}{cc} R & I\\ I & Z \end{array}\right]\geq 0. \end{array}$$

Then, the following algorithm is presented to get the maximum *η*_max⁡_.

**Algorithm 7**

 

*Step 1*. Choose a sufficiently small initial *η* > 0 such that there exists a feasible solution to (16), (19), and (23). Set *η*_max⁡_ = *η*.

* Step 2*. Find a feasible set (*P*_0_, *L*_0_, *W*_0_, *S*_0_, *Z*_0_, *R*_0_, *U*_0_, *H*_0_, *V*) satisfying (16), (19), and (23).

* Step 3*. Solve the following LMI problem for the variables (*P*, *L*, *W*, *S*, *Z*, *R*, *U*, *H*, *V*):

 Minimize tr{*LP*_*k*_ + *L*_*k*_ *P* + *UH*_*k*_ + *U*_*k*_ *H* + *RZ*_*k*_ + *R*_*k*_ *Z*}
 subject to (16), (19) and (23).

Set *P*_*k*+1_ = *L* ^−1^, *L*_*k*+1_ = *L*, *U*_*k*+1_ = *U*, *H*_*k*+1_ = *U* ^−1^, *R*_*k*+1_ = *R*, and *Z*_*k*+1_ = *R* ^−1^.

*Step 4.* If LMIs (6) and (7) are feasible with a given *K* derived in Step 3 for the variables (*P*, *Q*, *Z*, *G*), then set *η*_max⁡_ = *η* and return to Step 2 after increasing *η* to some extent. If LMIs (6) and (7) are infeasible within a specified number of iterations, then exit. Otherwise, set *k* = *k* + 1 and go to Step 3.

**Remark 8**

In [14, 16], the iteration condition is *P*_*k*+1_ = *P*, *L*_*k*+1_ = *L*, *U*_*k*+1_ = *U*, *H*_*k*+1_ = *H*, *R*_*k*+1_ = *R*, and *Z*_*k*+1_ = *Z*. Note that step 3 in Algorithm 7 is different from those in [14, 16]. Considering that *H* = *U* ^−1^, *P* = *L* ^−1^, *Z* = *R* ^−1^, we replace the above iteration condition with *P*_*k*+1_ = *L* ^−1^, *L*_*k*+1_ = *L*, *U*_*k*+1_ = *U*, *H*_*k*+1_ = *U* ^−1^, *R*_*k*+1_ = *R*, and *Z*_*k*+1_ = *R* ^−1^. The new iteration condition will help in reducing the iteration number and the amount of computations, which is verified by the numerical example in the next section.

## 4. Numerical Example

In this section, we give the following numerical example to illustrate the effectiveness of the proposed method.

**Example 1**

Consider system (1) with

(24) $$\begin{array}{c} A=\left[\begin{array}{cc} 0 & 1\\ 1 & -2 \end{array}\right],\;\;B=\left[\begin{array}{c} 1\\ 0 \end{array}\right],\;\;C=\left[\begin{array}{cc} 1 & -0.5 \end{array}\right],\\ D=\left[\begin{array}{c} 0\\ 1 \end{array}\right],\;\;\varphi \left(\cdot \right)\in F\left[0,1\right]. \end{array}$$

This example has been discussed in [15, 16]. It is reported that the closed-loop system (3) is stable for *η* = 1.2841 with *K* = [−0.5324 −0.2419] in [15] and *η* = 1.5250 with *K* = [−0.5347 −0.2469] after 84 iterations in [16]. By applying Algorithm 7, the MADB is *η* = 1.5279 with *K* = [−0.5296 −0.2532] after 39 iterations. Clearly, the method proposed in this paper is better than those in [15, 16].

## 5. Conclusion

This paper has investigated the problem of absolute stability and stabilization of Lurie NCSs. A new absolute stability condition of networked closed-loop system with a given controller gain matrix has been first established by employing a reciprocally convex technique to consider the relationship between the network-induced delay and its varying interval. Then, based on the resulting condition, a networked state feedback controller has been proposed by employing an improved CCL algorithm with a new iteration condition. Finally, a numerical example has been given to show the improvement of the proposed method.
